# Risk Factors for the Prognosis of Endometrioid Endometrial Cancer: A Chinese Cohort Study

**DOI:** 10.1155/ogi/9931547

**Published:** 2026-06-18

**Authors:** Xiaoyi Bai, Zidan Lin, Suiying Liang, Chun Bian, Jiemei Hu

**Affiliations:** ^1^ Department of Gynecology, Guangdong Provincial People’s Hospital (Guangdong Academy of Medical Sciences), Southern Medical University, Guangzhou, China, fimmu.com

**Keywords:** endometrioid endometrial carcinoma, FIGO stage, prognosis, survival analysis, tumor size

## Abstract

**Objective:**

To identify independent prognostic factors for endometrioid endometrial carcinoma based on a 10‐year follow‐up Chinese cohort study.

**Methods:**

This retrospective study included 142 patients diagnosed with endometrioid endometrial carcinoma from 2009 to 2013. Clinical, pathological, and immunohistochemical data were analyzed. Patients were categorized into three outcome groups: no recurrence, recurrence with ongoing survival, and death group. Kaplan–Meier survival analysis and univariate and multivariate Cox proportional hazards regression analysis were conducted to assess disease‐free survival (DFS) and overall survival (OS).

**Results:**

Advanced FIGO stage and tumor size ≥ 4 cm were independent predictors of poorer OS. Grade 3 histology also independently correlated with reduced survival. For DFS, tumor size ≥ 3 cm and advanced stage were significant risk factors. Progesterone receptor (PR) positivity and lack of lymphovascular space invasion (LVSI) were significantly associated with better prognosis in univariate analysis but were not retained in multivariate models.

**Conclusions:**

Tumor size and FIGO stage are key prognostic factors for recurrence and mortality in endometrioid endometrial carcinoma. Grade 3 histology further worsens the prognosis. Traditional pathological markers continue to be critical for individualized risk assessment and treatment planning.

## 1. Introduction

Endometrial carcinoma (EC) is the most common malignant tumor of the female reproductive tract in Western and other developed countries. Its incidence has continued to increase by 1% per year since the mid‐2000s [1]. Meanwhile, both its incidence and mortality rates are steadily increasing among Chinese women [2]. EC is typically diagnosed after menopause, with approximately 70% of cases detected at an early stage. These early‐stage patients generally have a favorable prognosis, with a 5‐year overall survival (OS) rate exceeding 70% [1, 3–5]. Endometrioid endometrial adenocarcinoma represents the most prevalent histological subtype, and its prognosis is generally better than that of other aggressive variants such as serous and clear cell ECs. However, the mortality of EC has increased more rapidly than the incidence rate, approximately resulting in more than 13,000 deaths in the United States in 2025 [1].

The primary treatment for EC is surgical staging, which includes total hysterectomy (TH) and bilateral salpingo‐oophorectomy (BSO), often combined with pelvic and/or para‐aortic lymphadenectomy depending on risk stratification [6]. Adjuvant therapy is administered to intermediate‐ or high‐risk patients based on a combination of clinicopathological features, International Federation of Gynecology and Obstetrics (FIGO) stage, tumor grade, etc. [4, 5, 7].

The Cancer Genome Atlas (TCGA) classified patients with EC into four major molecular subtypes with differing clinical prognoses: (I) DNA polymerase epsilon (POLE) mutations, (II) microsatellite instability‐high (MSI‐H), (III) copy number‐low (wild‐type p53), and (IV) copy number‐high (abnormal p53) [8]. Research has found that molecular classification is effective in predicting prognosis and selecting surgery type and adjuvant therapy; therefore, ESGO/ESTRO‐/ESP guidelines and Chinese expert consensus recommend incorporating molecular classification into FIGO staging and guiding patient management [9, 10]. However, molecular classification tests are much more complex and expensive which limits their universal application. What’s more, risk stratifications based on molecular classification are mainly clear in those with the POLE mutation or p53 abnormality, which only comprise a minority of EC.

On the contrary, traditional risk stratifications are easier and cheaper to test in clinical routines, and they also provide effective information for patients’ management after surgery. Previous studies have identified several prognostic risk factors in EC, including age, histological subtype and grade, FIGO stage, tumor size, and lymphovascular space invasion (LVSI), among others [11]. However, these factors remain insufficient to accurately predict clinical outcomes in many patients.

To address this gap, we conducted a long‐term follow‐up study of possurgical EC patients in a Chinese cohort over a period exceeding 10 years. Our objective was to identify independent prognostic factors specifically associated with endometrioid EC, with the aim of improving risk stratification and informing personalized treatment strategies.

## 2. Materials and Methods

### 2.1. Study Cohort

This was a retrospective cohort study conducted at Guangdong Provincial People’s Hospital. Patients diagnosed with endometrial cancer following surgery between March 2009 and February 2013 were consecutively recruited. Written informed consent was obtained from all participants, and patients were followed for a period of 10 years. Patients underwent staging surgery with TH/BSO and lymph node assessment via a laparoscopic or abdominal route. Patients meeting Mayo criteria (MI < 50%, tumor size < 2 cm, and Grade 1 or 2) were exempt from pelvic lymphadenectomy [12], while others underwent pelvic lymph node dissection, with or without para‐aortic lymph node dissection. The tumor size was assessed by measuring by operator in the specimen after hysterectomy. Postoperative adjuvant therapy was administered to intermediate‐ and high‐risk patients based on established risk stratification criteria. During follow‐up, recurrence status and timing, as well as survival status and survival duration, were recorded. Tumor recurrence was confirmed by clinical examination, imaging, or histopathological analysis, either during routine surveillance or upon presentation of symptoms such as vaginal bleeding or abdominal pain. Comprehensive clinical data, including age at diagnosis, menopausal status, gravida, parity, surgical details, histopathological characteristics, and clinical staging, were extracted from the hospital’s electronic medical records. Patients were excluded if they had nonendometrioid histological subtypes, had received radiotherapy, chemotherapy, or hormone therapy before surgery, had a history of other malignant tumors, or were lost to follow‐up. The study protocol was approved by the Research Ethics Committee of Guangdong Provincial People’s Hospital, Guangdong Academy of Medical Sciences (Approval No. GDREC 2016370H), in accordance with the Declaration of Helsinki.

### 2.2. Histopathological Assessment

Tumor subtype was classified according to the WHO International Histological Classification of Tumors. Myometrial invasion (MI) was categorized as superficial (< 50%) or deep (≥ 50%), and LVSI was defined as the presence of tumor cells within or adherent to the wall of blood vessels or lymphatic channels. Clinical staging was determined according to the 2009 FIGO classification system via clinicopathological assessment [13]. Formalin‐fixed paraffin‐embedded tissue sections after hysterectomy were used to evaluate progesterone receptor (PR), estrogen receptor (ER), p53, Bcl‐2, vascular endothelial growth factor (VEGF), CA125, and Ki‐67 expression via immunohistochemical (IHC) staining. ER, PR, Bcl‐2, VEGF, and CA125 expression were determined by the percentage of positive nuclei within the tumor area, and markers were defined as negative status if their percentage were ≤ 10%; otherwise, they were positive. Wild‐type p53 staining was characterized by an admixture of negative cells and weakly and strongly positive cells, while overexpression and complete absence were interpreted as aberrant type. Ki‐67 was calculated by the percentage of nucleic‐positive cells to all tumor cells.

### 2.3. Statistical Analysis

Based on long‐term outcomes, patients were classified into three groups: no recurrence (Group 1), recurrence with ongoing survival (Group 2), and death group (Group 3) during the follow‐up period. Continuous variables were expressed as medians with interquartile ranges (IQRs), while categorical variables were summarized as counts and percentages. Mann–Whitney *U* tests were used for two‐group comparisons and Kruskal–Wallis tests with Bonferroni post hoc correction for comparisons across the three groups for continuous variables. Categorical variables were analyzed using chi‐square tests or Fisher’s exact tests, as appropriate.

OS was defined as the interval from the date of diagnosis to cancer‐related death or last follow‐up. Disease‐free survival (DFS) was defined as the interval from the date of diagnosis to the earliest occurrence of relapse or last follow‐up without recurrence. Survival outcomes were assessed using Kaplan–Meier survival analysis and compared with log‐rank tests. A univariate Cox proportional hazards regression model was used to identify potential indicators associated with OS and DFS. We included those significant variables in univariate Cox regression with *p* < 0.05 into multivariate Cox regression. Forward stepwise COX regressions were used to simplify the model and avoid overfitting. Hazard ratios (HRs) and corresponding 95% confidence intervals (CIs) were reported. A two‐sided *p* value < 0.05 was considered statistically significant. All analyses were conducted using SPSS version 26.0 (IBM Corp., Armonk, NY, USA).

## 3. Results

### 3.1. Patient Characteristics

**FIGURE 1:**
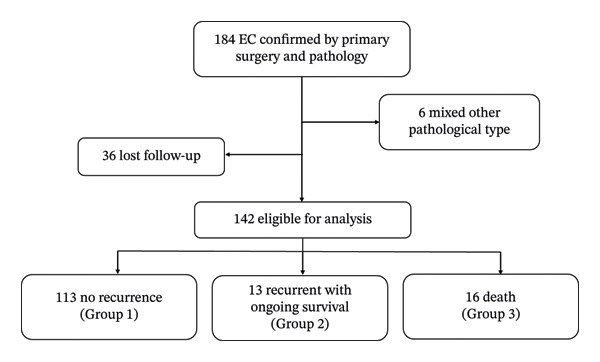
Flow diagram of patient enrollment and group allocation in the study.

A total of 184 patients with EC met the inclusion criteria during the study period. Among them, six cases with mixed histological subtypes and 36 cases lost to follow‐up were excluded, leaving 142 eligible patients for final analysis (Figure 1). The tumor size, histologic grade, clinical stage, and parity were not significantly different between included and lost follow‐up patients except for patients’ age (Supporting Table 1). The median age at diagnosis was 55.0 years (interquartile range [IQR], 49.0–60.0), with no statistically significant difference among the three outcome groups. Eight patients (5.6%) had never been pregnant, and 11 (7.7%) had no parity; the distributions of gravida and parity were comparable across groups. The majority of patients (*n* = 86, 60.5%) were postmenopausal, with a median time from menopause to disease onset of 8.5 years (IQR, 4.0–13.0). Most patients (*n* = 79, 55.6%) underwent open laparotomy, and the surgical approach did not differ significantly among outcome groups.

### 3.2. Clinical Outcomes and Pathological Results

The median tumor size in Group 3 was 5.0 cm (IQR, 3.62–5.88), which was significantly larger than that in Group 1 (2.0 cm, IQR 1.0–3.5, *p* < 0.001) and numerically larger than in Group 2 (3.0 cm, IQR 2.0–4.75), though the latter difference did not reach statistical significance. Nearly all patients (93.8%) in Group 3 underwent lymphadenectomy, which was significantly higher in Group 2 (76.9%) and Group 1 (52.2). Also, adjuvant treatment after surgery was applied to most patients in Group 3 (81.3%) according to the pathological results. The recurrence interval was significantly shorter in Group 3 (median 18.5 months) than in Group 2 (median 47.0 months), with all Group 3 patients having distant recurrence while only a small portion (15.4%) of Group 2 patients relapsed distantly. Chemotherapy or combined therapy was used in distant recurrence, while radiotherapy was applied to most local recurrence.

Regarding IHC markers, the absence of PR expression was significantly more frequent in Group 3 than in Group 1 (*p* < 0.05), while no significant differences were observed among groups for ER, p53, Bcl‐2, VEGF, CA125, or Ki‐67 expression. LVSI was significantly more commonly present in Group 3 compared to Group 1 (*p* < 0.001), with no significant difference between Group 1 and Group 2 or between Group 2 and Group 3. The proportion of Grade 3 (G3) tumors was higher in Group 3 (*n* = 6, 37.5%) than in Groups 1 and 2, though the difference did not reach statistical significance. Only 4 patients (25.0%) in Group 3 were classified as FIGO Stage I, which was significantly lower than 100 patients (88.5%) in Group 1 and 9 patients (69.2%) in Group 2 (*p* < 0.001) (Table 1).

**TABLE 1:** Clinicopathological characteristics.

Characteristics	Group 1 (*n* = 113)	Group 2 (*n* = 13)	Group 3 (*n* = 16)	*p*
Age (years)	54.0 (48.5–60.0)	56.0 (55.0–60.0)	54.5 (49.8–59.3)	0.155
Null gravida	5 (4.4)	0 (0.0)	3 (18.8)	0.090^#^
Null parity	7 (6.2)	1 (7.7)	3 (18.8)	0.135^#^
Menopause				
No	45 (39.8)	4 (30.8)	7 (43.8)	0.763
Yes	68 (60.2)	9 (69.2)	9 (56.3)	
Menopause age (years)	50.0 (47.2–52.0)	50.0 (50.0–52.5)	48.0 (44.5–51.0)	0.463
Menopause‐occurrence interval (years)	7.5 (4.0–13.0)	6.0 (4.5–13.0)	10.0 (1.5–12.5)	0.941
Surgical type				
Laparotomy	63 (55.8)	7 (53.8)	9 (56.3)	0.990
Laparoscopic	50 (44.2)	6 (46.2)	7 (43.7)	
Size (cm)	2.0 (1.0–3.5)^∗^	3.0 (2–4.8)	5.0 (3.6–5.9)^∗^	0.035
Lymphadenectomy	59 (52.2)^∗&^	10 (76.9)^∗^	15 (93.8)^&^	0.003
Adjuvant treatment after surgery	28 (24.8)^∗^	5 (38.5)	13 (81.3)^∗^	< 0.001
Recurrence time (months)	N.A	47.0 (19.0–69.5)	18.5 (16.0–43.3)	0.025
Recurrence type				
Distant	N.A	2 (15.4)	16 (100.0)	< 0.001
Postrecurrence therapy				
Radiotherapy	N.A	9 (69.2)	2 (12.5)	< 0.001
Chemotherapy	N.A	0 (0.0)	10 (62.5)	
Surgery	N.A	1 (7.7)	0 (0.0)	
Combined	N.A	3 (23.1)	4 (25.0)	
PR				
−	7 (6.2)^∗^	1 (7.7)	4 (25.0)^∗^	0.039^#^
+	106 (93.8)	12 (92.3)	12 (75.0)	
ER				
−	9 (8.0)	2 (15.4)	2 (12.5)	0.416^#^
+	104 (92.0)	11 (84.6)	14 (87.5)	
P53				
Wild type	105 (92.9)	12 (92.3)	13 (81.3)	0.212^#^
Aberrant	8 (7.1)	1 (7.7)	3 (18.7)	
Bcl2				
−	42 (37.2)	7 (53.8)	7 (43.8)	0.473
+	71 (62.8)	6 (46.2)	9 (56.2)	
VEGF				
−	18 (15.9)	2 (15.4)	3 (18.8)	0.916^#^
+	95 (84.1)	11 (84.6)	13 (81.2)	
CA125				
−	6 (5.3)	0 (0.0)	0 (0.0)	0.99^#^
+	107 (94.7)	13 (10.00)	16 (100.0)	
Ki 67 (%)	30.0 (10.0–60.0)	30.0 (17.5–55.0)	45.0 (10.0–70.0)	0.786
LVSI				
Absent	102 (91.1)	11 (84.6)	8 (50.0)	< 0.001
Present	10 (8.9)^∗^	2 (15.4)	8 (50.0)^∗^	
Grade				
G1	73 (64.6)	9 (69.2)	8 (50.0)	0.404
G2	22 (19.5)	2 (15.4)	2 (12.5)	
G3	18 (15.9)	2 (15.4)	6 (37.5)	
Clinical stage (FIGO 2009)				
I	100 (88.5)^∗&^	9 (69.2)^∗^	4 (25.0)^&^	< 0.001
II	9 (8.0)	0 (0.0)	1 (6.3)	
III	4 (3.5)	4 (30.8)	10 (62.5)	
IV	0 (0.0)	0 (0.0)	1 (6.3)	

*Note:* Group 1, no recurrence; Group 2, recurrence with ongoing survival; Group 3, death group. −, negative; +, positive. LVSI, lymphovascular space invasion; FIGO, International Federation of Gynaecology and Obstetrics.

Abbreviations: CA125, cancer antigen 125; ER, estrogen receptor; N.A., not applicable; PR, progesterone receptor; VEGF, vascular endothelial growth factor.

^∗,&^two groups with the same superscript were significantly different between two groups with post hoc test.

^#^Fisher’s exact test.

### 3.3. Prognostic Factors for OS

Kaplan–Meier survival curves revealed that several clinicopathological factors significantly influenced OS in patients with endometrioid EC. Patients with tumor size ≥ 4 cm had significantly poorer survival outcomes compared to those with smaller tumors (*p* < 0.001). Similarly, negative PR expression (*p* = 0.006) and the presence of LVSI (*p* < 0.001) were strongly associated with decreased OS. Furthermore, advanced FIGO stage was a strong predictor of poor OS, with higher‐stage patients exhibiting markedly reduced survival compared to those diagnosed at Stage I (*p* < 0.001) (Figure 2).

FIGURE 2Kaplan–Meier survival curve of prognostic factors for overall survival. (A) Tumor size, (B) progesterone receptor, (C) FIGO stage, (D) lymphovascular space invasion, and (E) histological grade.

**Figure:**
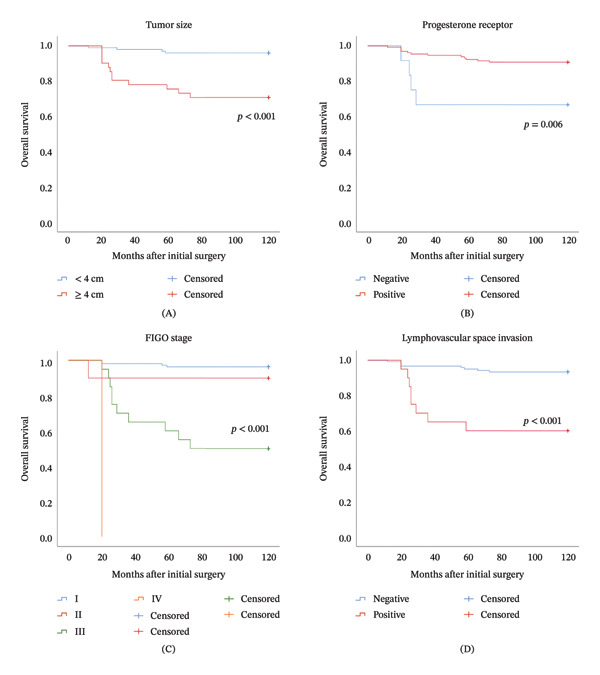

**Figure:**
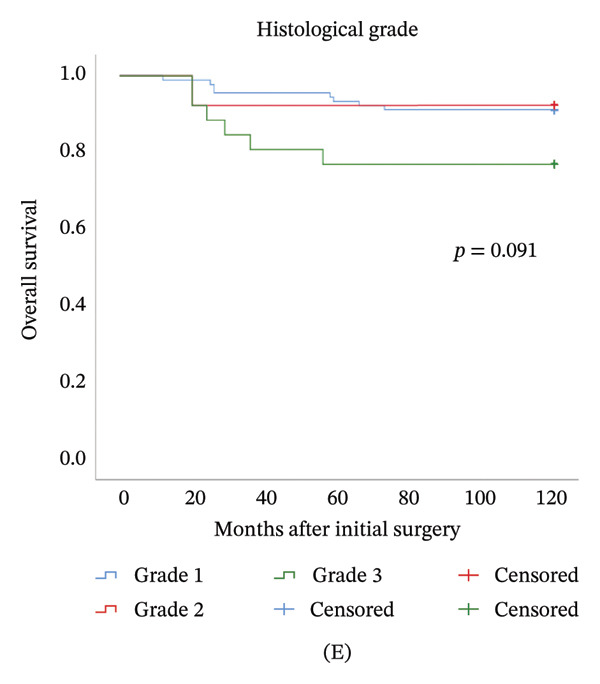

Univariate Cox regression analysis identified tumor size ≥ 4 cm, LVSI, G3 histology, and advanced FIGO stage (Stages III‐IV) as significant risk factors for disease‐specific mortality. Conversely, positive PR expression was associated with improved survival. In the multivariate Cox regression model, advanced FIGO stage emerged as the most potent independent predictor of poor OS (HR = 10.94, *p* < 0.001), followed by tumor size ≥ 4 cm (HR = 5.81, *p* = 0.005) and G3 histology (HR = 3.93, *p* = 0.015) (Table 2).

**TABLE 2:** Univariate and multivariate COX regression analysis of prognostic factors for overall survival.

Factors	Univariate analysis		Multivariate analysis	
	HR (95% CI)	*p*	HR (95% CI)	*p*
Tumor Size (≥ 4 cm)	8.42 (2.71–26.12)	< 0.001	5.81 (1.69–19.96)	0.005
PR positive	0.24 (0.08–0.73)	0.013	—	—
LVSI	7.28 (2.72–19.48)	< 0.001	—	—
Grade 3 (reference: Grades 1 and 2)	2.92 (1.06–8.05)	0.038	3.93 (1.31–11.76)	0.015
Advanced Stage (reference: Stages I and II)	18.93 (6.53–54.87)	< 0.001	10.94 (3.54–33.85)	< 0.001

*Note:* LVSI, lymphovascular space invasion.

Abbreviations: HR, hazard ratio; PR, progesterone receptor.

### 3.4. Prognostic Factors for DFS

In terms of DFS, Kaplan–Meier survival curves showed that patients with tumors ≥ 3 cm experienced significantly higher recurrence rates than those with smaller tumors (*p* < 0.001). Negative PR expression (*p* = 0.04) and the presence of LVSI (*p* < 0.001) were also significantly associated with increased recurrence risk. Advanced FIGO stage remained a critical factor, with higher‐stage patients demonstrating substantially reduced DFS compared to those with Stage I disease (*p* < 0.001) (Figure 3).

FIGURE 3Kaplan–Meier survival curve of prognostic factors for disease‐free survival. (A) Tumor size, (B) progesterone receptor, (C) FIGO stage, (D) lymphovascular space invasion, and (E) histological grade.

**Figure:**
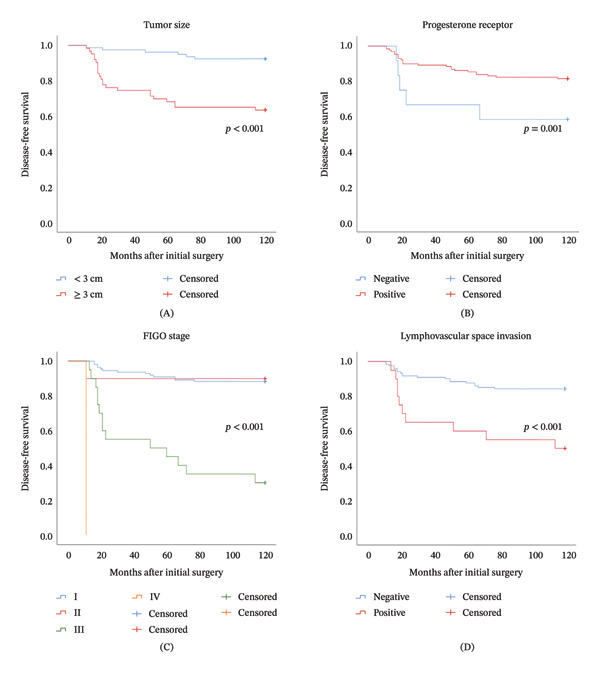

**Figure:**
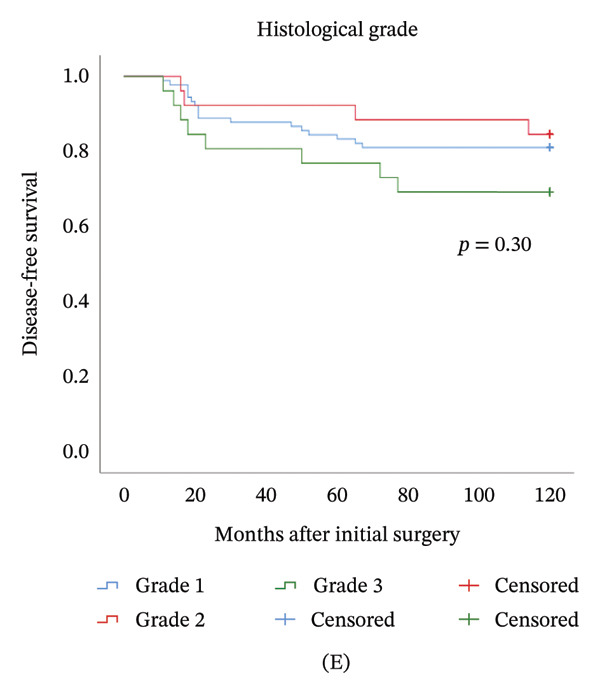

For disease recurrence, univariate analysis revealed tumor size ≥ 3 cm, presence of LVSI, and advanced FIGO stage as significant risk factors, while positive PR expression remained a protective factor. Multivariate analysis further confirmed that advanced stage (HR = 9.76, *p* < 0.001) and tumor size ≥ 3 cm (HR = 4.61, *p* = 0.001) were independently associated with increased risk of recurrence (Table 3).

**TABLE 3:** Univariate and multivariate COX regression analysis of prognostic factors for disease‐free survival.

Factors	Univariate analysis		Multivariate analysis	
	HR (95% CI)	*p*	HR (95% CI)	*p*
Tumor Size (≥ 3 cm)	5.84 (2.37–14.35)	< 0.001	4.61 (1.83–11.63)	0.001
PR positive	0.38 (0.15–0.99)	0.049	—	—
LVSI	3.90 (1.81–8.39)	0.001	—	—
Grade 3 (reference: Grades 1 and 2)	1.85 (0.82–4.17)	0.140	—	—
Advanced stage (reference: Stages I and II)	11.43 (5.46–23.96)	< 0.001	9.76 (4.52–21.07)	< 0.001

*Note:* LVSI, lymphovascular space invasion.

Abbreviations: HR, hazard ratio; PR, progesterone receptor.

## 4. Discussion

In this study, we identified several clinicopathological factors associated with recurrence and survival in patients with endometrioid EC. Tumor size and FIGO stage emerged as independent prognostic factors for both DFS and OS, while Grade 3 histology was independently associated with poor OS. PR status and LVSI were also significantly related to prognosis in univariate analyses, though they did not retain significance in multivariate models.

Although tumor size is not currently incorporated into routine FIGO staging or postoperative adjuvant therapy algorithms, increasing evidence suggests its importance in endometrial cancer prognosis. Prior studies have reported that larger tumor size is associated with deep MI, lymph node metastasis, and increased risk of local or distant recurrence [14–18]. Various cutoff values (2 cm, 3 cm, or 4 cm) have been proposed in previous studies, although a unified standard is lacking. In our cohort, we found that tumor diameter > 3 cm was significantly associated with disease recurrence, while diameter > 4 cm independently predicted disease‐specific mortality. These findings support tumor size as a valuable parameter for individualized risk assessment in EC.

PR expression plays a critical role in endometrial physiology and cancer progression. Progesterone acts as a functional antagonist of estrogen by promoting differentiation and inhibiting proliferation of endometrial cells [19, 20]. The expression of PR is typically reduced in EC compared to atypical hyperplasia and normal endometrial tissue [21], and PR negativity is often associated with higher tumor grade, lymph node involvement, advanced FIGO stage, nonendometrioid histology, and LVSI [22–25]. Furthermore, PR expression is known to reflect tumor differentiation status, as it tends to decline in poorly differentiated tumors [21]. Progesterone or progestin therapies inhibit tumor growth, reduce invasiveness, and promote apoptosis [26, 27]. PR status has also been proposed as a potential marker to guide lymphadenectomy in early‐stage EC [22, 28, 29]. Our findings align with these previous studies: PR positivity was associated with improved DFS and OS in univariate analysis and remained an independent predictor for OS in the multivariate model. Notably, the recent OATH trial further highlighted the therapeutic relevance of PR, demonstrating the efficacy of combined antiprogesterone (onapristone) and antiestrogen (anastrozole) treatment in hormone receptor‐positive cases [30].

LVSI, defined as the presence of tumor cells within lymphatic or blood vessels, has long been considered a marker of aggressive tumor biology. A large population‐based study identified LVSI as the strongest independent predictor of lymph node metastasis and poor prognosis in endometrioid adenocarcinoma. Importantly, even in node‐negative patients, LVSI positivity is associated with reduced survival, potentially due to hematogenous dissemination [31]. Jorge et al. similarly reported that LVSI is independently predictive of lymph node metastasis and poor survival outcomes in early‐stage EC [32]. In recognition of its prognostic significance, the 2023 FIGO staging update now incorporates LVSI, distinguishing between focal and substantial involvement within Stage I and assigning extensive LVSI to Stage II [33–35]. In our study, LVSI was present in 50% of deceased cases compared to only 8.9% in the no‐recurrence group, and it was significantly associated with recurrence and OS in univariate analysis. However, it was not significant in multivariate analysis because it had significant correlations with FIGO staging and tumor size in our cohort (data not shown). Our study had a small sample size, so it was eliminated by multivariate regression analysis through variable selection. But we still could conclude that LVSI was a potential predictor for the prognosis of EC.

Histological grade remains a fundamental parameter in EC risk classification. Grades 1 and 2 are generally categorized as low grade, while Grade 3 is considered high grade. Numerous studies have shown that G3 tumors are associated with worse outcomes [36, 37]. Mariani et al. identified G3 as the strongest predictor of vaginal recurrence [38], and the PORTEC‐1 trial confirmed its association with higher locoregional recurrence rates in multivariate analysis [39]. Our study corroborated these findings, with G3 histology serving as an independent predictor of poor OS.

The prognostic value of FIGO stage remains unequivocal. Early‐stage disease accounts for approximately 70% of EC cases and is associated with excellent prognosis, with 5‐year OS rates exceeding 90% in low‐grade endometrioid tumors [40]. In contrast, Stages III and IV disease show markedly reduced survival, with 5‐year OS rates of 60%–70% and less than 20%, respectively [41, 42]. Our cohort showed comparable patterns: early‐stage patients had a recurrence rate of 11% and OS of 95%, while advanced‐stage patients had a recurrence rate of 79% and OS of 52%. FIGO stage was the most significant predictor of both DFS and OS in multivariate models.

This study has several strengths. The cohort was recruited over a short period, minimizing treatment variability. Long‐term follow‐up of 10 years ensured meaningful survival data. We also collected a wide array of clinical and pathological variables, enabling a comprehensive assessment of prognostic factors. However, limitations include its single‐center design and the small number of adverse outcomes, which may reduce statistical power. Additionally, molecular classification has been recommended for risk stratification and postsurgery management since 2013; however, our cohort was recruited from 2009 to 2013, and molecular classification was not available in this cohort. In clinical practice, we encourage patients to do molecular classification tests, and we hope to incorporate them in future studies. Also, a future larger prospective cohort study to validate the different tumor size cut‐offs in the current study for prognosis of DFS and OS and how they overlap with molecular classification is needed.

In conclusion, our findings suggest that larger tumor size and advanced FIGO stage are independent risk factors for both recurrence and mortality in endometrial cancer, while Grade 3 histology is an independent predictor of poor survival. Traditional pathological variables continue to play a pivotal role in prognosis and treatment decisions, and future studies incorporating molecular classification may further refine risk assessment in this disease.

## Author Contributions

Xiaoyi Bai did data collection, data interpretation, and manuscript drafting.

Zidan Lin was responsible for data analysis, statistical interpretation, and critical revision of the manuscript.

Suiying Liang participated in patient recruitment, clinical data acquisition, and follow‐up coordination.

Chun Bian contributed to the literature review, figure/table preparation, and manuscript formatting.

Jiemei Hu contributed to the conception and design of the study and manuscript drafting.

## Funding

No funding was received for this manuscript.

## Conflicts of Interest

The authors declare no conflicts of interest.

## Supporting Information

Additional supporting information can be found online in the Supporting Information section.

## Supporting information


**Supporting Information** Supporting Table 1. Characteristics between included and lost follow‐up patients.

## Data Availability

The data that support the findings of this study are available on request from the corresponding author. The data are not publicly available due to privacy or ethical restrictions.
