# Supplementary material for: Risk Factors for the Prognosis of Endometrioid Endometrial Cancer: A Chinese Cohort Study
Source: Obstet Gynecol Int. 2026 Jun 18;2026:9931547. doi: 10.1155/ogi/9931547 (PMC13278023; doi:10.1155/ogi/9931547)
Supplement: Supplementary file 1 — Supporting Information Supporting Table 1. Characteristics between included and lost follow‐up patients. [file OGI-2026-9931547-s001.docx]

Supplementary Table 1. Characteristics between included and lost follow-up patients

| Characteristics | Included  (n=142) | Lost follow-up (n=36) | *P* |
| --- | --- | --- | --- |
| Age (years) | 55.0 (49.0-60.0) | 51.5 (43.8-55.0) | 0.013 |
| Null Gravida | 8 (5.6) | 2 (5.5) | 0.985 |
| Null Parity | 11 (7.7) | 2 (5.5) | 0.652 |
| Tumor Size (cm) | 2.5 (1.0-4.0) | 2.0 (1.0-3.5) | 0.385 |
| Grade |  |  |  |
| G1 | 90 (60.4) | 25 (69.4) | 0.768 |
| G2 | 26 (18.3) | 6 (16.7) |  |
| G3 | 26 (18.3) | 5 (13.9) |  |
| Clinical stage (FIGO 2009) |  |  |  |
| I | 113(79.6) | 28 (77.8) | 0.075 |
| II | 10 (7.0) | 3 (8.3) |  |
| III | 18 (12.6) | 2 (5.6) |  |
| IV | 1 (0.7) | 3 (8.3) |  |

*FIGO*, international federation of gynaecology and obstetrics.
